# Transforming Clinical Data into Actionable Prognosis Models: Machine-Learning Framework and Field-Deployable App to Predict Outcome of Ebola Patients

**DOI:** 10.1371/journal.pntd.0004549

**Published:** 2016-03-18

**Authors:** Andres Colubri, Tom Silver, Terrence Fradet, Kalliroi Retzepi, Ben Fry, Pardis Sabeti

**Affiliations:** 1 Center for Systems Biology, Department of Organismic and Evolutionary Biology, Harvard University, Cambridge, Massachusetts, United States of America; 2 Broad Institute of MIT and Harvard, Cambridge, Massachusetts, United States of America; 3 Harvard College, Cambridge, Massachusetts, United States of America; 4 Fathom Information Design, Boston, Massachusetts, United States of America; 5 Department of Psychiatry, Massachusetts General Hospital, Boston, Massachusetts, United States of America; 6 Department of Immunology and Infectious Disease, Harvard School of Public Health, Boston, Massachusetts, United States of America; Imperial College London, UNITED KINGDOM

## Abstract

**Background:**

Assessment of the response to the 2014–15 Ebola outbreak indicates the need for innovations in data collection, sharing, and use to improve case detection and treatment. Here we introduce a Machine Learning pipeline for Ebola Virus Disease (EVD) prognosis prediction, which packages the best models into a mobile app to be available in clinical care settings. The pipeline was trained on a public EVD clinical dataset, from 106 patients in Sierra Leone.

**Methods/Principal Findings:**

We used a new tool for exploratory analysis, *Mirador*, to identify the most informative clinical factors that correlate with EVD outcome. The small sample size and high prevalence of missing records were significant challenges. We applied multiple imputation and bootstrap sampling to address missing data and quantify overfitting. We trained several predictors over all combinations of covariates, which resulted in an ensemble of predictors, with and without viral load information, with an area under the receiver operator characteristic curve of 0.8 or more, after correcting for optimistic bias. We ranked the predictors by their F1-score, and those above a set threshold were compiled into a mobile app, *Ebola CARE* (Computational Assignment of Risk Estimates).

**Conclusions/Significance:**

This method demonstrates how to address small sample sizes and missing data, while creating predictive models that can be readily deployed to assist treatment in future outbreaks of EVD and other infectious diseases. By generating an ensemble of predictors instead of relying on a single model, we are able to handle situations where patient data is partially available. The prognosis app can be updated as new data become available, and we made all the computational protocols fully documented and open-sourced to encourage timely data sharing, independent validation, and development of better prediction models in outbreak response.

## Author Summary

We introduce a machine-learning framework and field-deployable app to predict outcome of Ebola patients from their initial clinical symptoms. Recent work from other authors also points out to the clinical factors that can be used to better understand patient prognosis, but there is currently no predictive model that can be deployed in the field to assist health care workers. Mobile apps for clinical diagnosis and prognosis allow using more complex models than the scoring protocols that have been traditionally favored by clinicians, such as Apgar and MTS. Furthermore, the WHO Ebola Interim Assessment Panel has recently concluded that innovative tools for data collection, reporting, and monitoring are needed for better response in future outbreaks. However, incomplete clinical data will continue to be a serious problem until more robust and standardized data collection systems are in place. Our app demonstrates how systematic data collection could lead to actionable knowledge, which in turn would trigger more and better collection, further improving the prognosis models and the app, essentially creating a virtuous cycle.

## Introduction

The 2014–15 EVD outbreak in West Africa has eclipsed in magnitude all combined past EVD outbreaks since the disease was first identified in 1976 [1]. As of February 17, 2016 (http://www.cdc.gov/vhf/ebola/outbreaks/2014-west-africa/case-counts.html), a total of 28,639 cases have been reported (15,251 laboratory-confirmed) and 11,316 total deaths. The outbreak constitutes one of the most serious worldwide health emergencies in modern times, with severe socioeconomic costs, particularly in the West African nations of Liberia, Sierra Leone, and Guinea. Although vaccine development is promising [2], the prospect of future outbreaks looms. The report of the WHO Ebola Interim Assessment Panel also points to several shortcomings in the initial response [3], noting that “better information was needed to understand best practices in clinical management” and that “innovations in data collection should be introduced, including geospatial mapping, mHealth communications, and platforms for self-monitoring and reporting”.

Given these circumstances, the development of accurate and accessible computational methods to track the progression of the outbreak and model various aspects of the disease is beneficial not only for the research community, but also for health care personnel in the field. In particular, prognosis prediction models based on the available patient information would be of great utility. Such predictive models can identify the clinical symptoms and laboratory results that should be tracked most closely during the onset of EVD, and give health care workers the ability to more accurately assess patient risk and therefore manage treatment more efficiently [4]. This data-driven prioritization could lead to higher recovery rates through stratified treatment [5], especially in resource-constrained areas, and would help doctors limit the evaluation of experimental EVD vaccines and treatments [6] with potentially harmful side effects only to highest-risk patients. These improvements in treatment, however, will only be achieved once larger datasets become available to overcome biases resulting from small samples.

Schieffelin et al. [7] presented the only publicly accessible, at the time of publication, clinical dataset from the West African EVD outbreak (available in various formats at http://fathom.info/mirador/ebola/datarelease) to enable clinical investigations. Although a large amount of very useful case and resource data has been made public throughout the outbreak (https://data.hdx.rwlabs.org/ebola), thanks to the efforts of numerous individuals and organizations, there is to our knowledge no other public source offering a similar level of clinical detail. The Schieffelin et al. dataset includes epidemiologic, clinical, and laboratory records of 106 patients treated at Kenema Government Hospital in Sierra Leone during the initial stages of the outbreak. The study also provides a simple heuristic to estimate mortality risk by defining an Ebola Prognostic Score (EPS), which predicts patient outcome based on symptom counts. EPS offers statistically significant differences between surviving and deceased patients with p < 0.001.

While data from other published clinical studies are not available, their summary results suggest that more advanced prognostic prediction models could be potentially useful to the field. Levine et al. [8] developed a diagnostics model using data from the Bong County Ebola Treatment Unit in Liberia, which predicts laboratory-confirmed EVD cases using six clinical variables. Yan et al. [9] carried out a multivariate analysis of 154 EVD patients from the Jui Government Hospital in Sierra Leone, and reported that age, fever, and viral load are independent predictors of mortality, while Zhang et al. [10] recently reported that age, chest pain, coma, confusion, and viral load are associated with EVD prognosis using a set of 63 laboratory-confirmed cases also from the Jui Government Hospital.

In this study, we employed the Schieffelin et al. EVD dataset to develop novel predictive models for patient prognosis, integrating a data-driven hypothesis making approach with a customizable Machine Learning (ML) pipeline, and incorporating rigorous imputation methods for missing data. We evaluated the predictors using a variety of performance metrics, identifying top predictors with and without viral load measurements, and packaged them into a mobile app for Android ad iOS devices (http://fathom.info/mirador/ebola/prognosis).

Our protocol exemplifies how data-driven computational methods can be useful in the context of an outbreak to extract predictive models from incomplete data, and to provide rapidly actionable knowledge to health workers in the field. Moreover, prognosis prediction software could complement ongoing efforts to develop rapid EVD diagnostics [11] and safe data-entry devices [12]. Given the availability of only one dataset from a single location, one Ebolavirus species (*Zaire ebolavirus*), and very specific time span and laboratory protocols, these models need to be interpreted in an exploratory sense and require further validation with independent clinical data from other EVD treatment sites [8] [9] [13] [14] [15]. We have made all of these resources publicly available and fully documented with the hope to encourage further methods development, independent validation, and greater data sharing in outbreak response.

## Methods

### Clinical dataset

**Fig 1:**
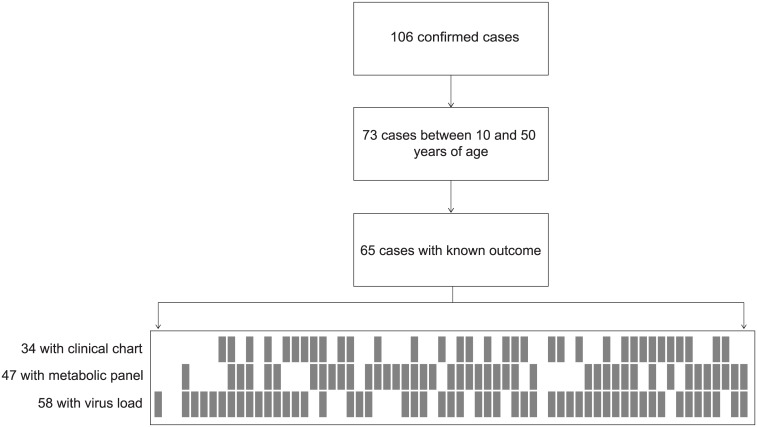
Case counts in the dataset. The flowchart indicates the total number of positive, confirmed EVD in the original dataset, from which we took only those corresponding to patients with ages between 10 and 50. From those, only 65 have known outcome and could be used for analysis. In the bottom part of this diagram, the numbers of cases within the last 65 that contain clinical chart (24), metabolic panel (47), and virus load data (58) are represented by fill rectangles. The resulting missing data pattern illustrates that only a few patients had known information across all categories.

Our analysis and modeling is based on the EVD clinical and laboratory data initially described by Schieffelin et al [7]. The Sierra Leone Ethics and Scientific Review Committee and the ethics committee at Harvard University have approved the study and public release of this clinical data, which has been de-identified to protect patient privacy. As indicated by Schieffelin, “these committees waived the requirement to obtain informed consent during the West African Ebola outbreak” and “all clinical samples and data were collected for routine patient care and for public health interventions.” The larger dataset comprises 213 suspected cases evaluated for Ebola virus infection at the Kenema Government Hospital (KGH) in Sierra Leone between May 25 and June 18, 2014. Outcome data was available for 87 of 106 Ebola-positive cases, giving a Case Fatality Rate (CFR) of 73% over the entire dataset. We considered 65 patients between 10 and 50 years of age. Within this group, not all individuals had complete clinical chart, metabolic panel, and virus load records available (Fig 1). Sign and symptom data were obtained at time of presentation on 34 patients that were admitted to KGH and had a clinical chart. Metabolic panels were performed on 47 patients with adequate sample volumes, with a Piccolo Blood Chemistry Analyzer and Comprehensive Metabolic Reagent Discs (Abaxis), following the manufacturer’s guidelines. Virus load was determined in 58 cases with adequate sample volumes using the Power SYBR Green RNA-to-CT 1-Step quantitative RT-PCR assay (Life Technologies) at Harvard University. Both metabolic panel and PCR data used to develop our models was collected during triaging of the patients upon admission, and follow-up data, although available for some patients, was not included in our analyses. We compiled this data into a single file in CSV format, and made it available in a public repository (http://dx.doi.org/10.5281/zenodo.14565), together with all original Excel spreadsheets and the cleaning and aggregation scripts (http://fathom.info/mirador/ebola/datarelease), as well as a Dataverse hosted on the Harvard Dataverse Network (http://dx.doi.org/10.7910/DVN/29296).

### Data-driven exploration: Mirador and MINE

In a separate effort, we designed the tool *Mirador* (http://fathom.info/mirador/) to allow users to identify statistical associations in complex datasets using an interactive visualization interface. This visual analysis is guided by an underlying statistical module that ranks the associations using pairwise Mutual Information [16]. *Mirador* automatically computes a sample estimate of the Mutual Information between each pair of variables inspected by the user, and performs a bootstrap significance test [17] to determine if the variables are independent within a confidence level set through the interface. This calculation relies on an optimal bin-width algorithm [18], which finds the grid minimizing the Mean Integrated Squared Error between the estimates from the data and the underlying joint distributions. The user can then rigorously test the hypothesis of association suggested by *Mirador* using more specialized tools such as R or SPSS, and finally incorporate them into predictive models. We used the Maximal Information Coefficient (MIC) statistic developed by Reshef et al [19], calculated with the MINE program (http://www.exploredata.net/), to rank the associations found with *Mirador*.

### Multiple Imputation of missing data

Since only 21 patients in the dataset contain complete clinical, laboratory, and viral load information, we applied three Multiple Imputation (MI) programs to impute the missing values: *Amelia II*, which assumes the data follows a multivariate normal distribution and uses a bootstrapped expectation-maximization algorithm to impute the missing values [20]; MICE [21] Multivariate Imputation by Chained Equations, where missing values in each variable are iteratively imputed given the other variables in the data until convergence is attained; and *Hmisc* [22], which is also based on the chained equations method. All MI methods require that the missing entries satisfy the Missing Completely At Random (MCAR) condition in order to generate unbiased results. Specifically, MCAR means that the distribution of the missing entries is entirely random and does not depend neither on the observed nor the missing values. Furthermore, *Amelia* requires the observed data to follow a multivariate normal distribution. We used Little’s MCAR chi-square test [23] and Jamshidian and Jalal's test for Homoscedasticity, Multivariate Normality, and MCAR [24] to rigorously test for these conditions.

After testing for the MCAR condition, we run each MI program *m* times to generate *m* “completed” copies of the original dataset, which we aggregated into a single training set of larger size (S4 Fig). We performed a detailed comparison of the performance of the predictor when using values imputed by each of the three MI programs, which is described in the results.

### Model training protocol

The ML pipeline takes as inputs the source data and a list of covariates, and outputs a trained predictor that can be evaluated with several accuracy metrics. It includes the following classifiers: a single-layer Artificial Neural Network (ANN) [25] implemented from scratch, and Logistic Regression (LR), Decision Tree (DT), and Support Vector Machine (SVM) classifiers from scikit-learn [26]. Each classifier was trained on all possible combination of input covariates, from the subset of found with *Mirador* and MINE, to avoid issues with variable selection methods [27], and to generate an ensemble of predictors that could be applied to different combinations of available clinical data.

We applied multiple cross-validation in order to train the classifiers for each selection of covariates. We first split the records without missing values into two sets with identical CFR, then set one aside for model testing. We combined the second set with the remaining records that include missing values, and used this data as the input for the MI programs. Depending on the percentage of complete records reserved for testing and the number of MIs, we ended up with testing sets of 6–10 cases and training sets of 200–300 cases. This ensured having more than 10 samples per variable during predictor training, the accepted minimum in predictive modeling [28]. We generated 100 of such testing/training set pairs by randomly reshuffling complete records between test set and training set.

### Model testing protocol

Each model was initially ranked by its mean F1-score, which is the weighted average of the precision and sensitivity. The mean and standard deviation were calculated over the 100 cross-validation iterations for each combination of input covariates. We then used the bootstrap method originally introduced by Harrell [29] to quantify the optimistic bias [30] in the area under the receiver operator curve (AUC or c-statistic). We generated 100 bootstrap samples with replacement for each model, and re-trained the model on these samples. We evaluated the AUC on the bootstrap sample and the original sample, and reported the mean of the AUC_boot_—AUC_orig_ difference as the estimated optimism.

Finally, we carried out standard logistic regression with variable selection, with the goal of evaluating the effect of our MI protocol on other model selection algorithms, and comparing the resulting standard model with the top-ranking models from our pipeline. We used the built-in step() function in R to perform backward variable selection with the Akaike Information Criterion (AIC), the ROCR package to compute AUC, and the Boot package to estimate of the optimistic bias with bootstrap sampling.

### Packaging predictive models as apps

**Fig 2:**
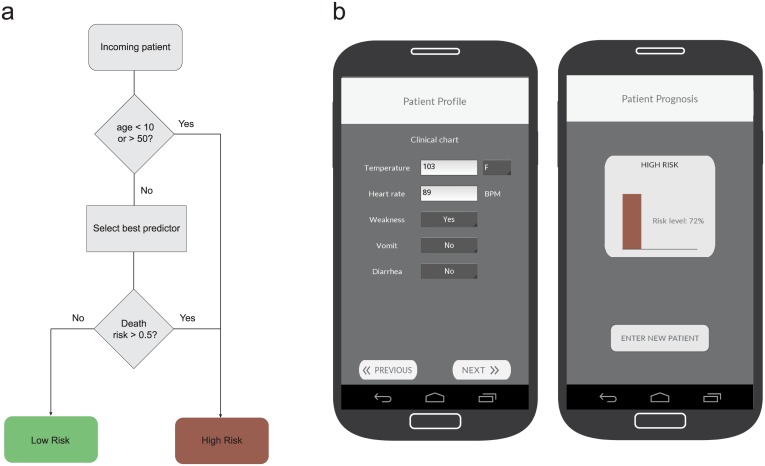
Prognosis-prediction protocol and app. The flowchart in panel A synthesizes the prognosis-prediction protocol that could be used in the field by application of the models obtained in this study. Incoming patients are classified either as low or high risk depending on their age and the output of the best predictor suitable for the available clinical symptoms; any patient under 10 or above 50 years of age is considered high risk. For patients in the 10–50 age range, the best predictor that includes the clinical symptoms of the patient, among those predictors with a mean F1-score above 0.9, is selected to make a risk prediction. The Ebola CARE app (panel b) implements this protocol in an easy to use interface, where the health care worker can enter the patient’s clinical information. The app automatically chooses the best model for the data, and returns the risk estimation.

The ML models generated by our pipeline are essentially Python scripts together with some parameter files. The Kivy framework (http://www.kivy.org) allowed us to package these scripts as mobile apps that can be deployed on tablets or smartphones through Google or Apple’s app stores. We created a prototype app including the models described in this paper, currently available as *Ebola CARE (Computational Assignment of Risk Estimates)*, shown in Fig 2. We have only implemented the ANN classifier into the *Ebola CARE* app for the time being, because the scikit-learn classifiers could not be compiled to run on Android devices, which is a requirement for our prognosis app. Once installed, the app is entirely stand-alone, does not require Internet connectivity to run, and can be updated once better models are available.

## Results

### Clinical factors correlated with EVD outcome

We began by identifying the clinical and laboratory factors that provide the strongest association with EVD outcome. Earlier reports indicate that EVD mortality rates in this outbreak are found to be significantly different among children [31] and older adults [7], and this pattern holds in our data: CFR is higher than 90% for the 18 patients older than 50 years of age, and 75% for the 14 patients under 10 years of age; we therefore restricted our analyses to patients between 10 and 50 years of age. Within this age range, exploratory analysis with *Mirador* (http://fathom.info/mirador/), led us to identify 24 clinical and laboratory factors that show plausible association with EVD outcome: virus load (PCR), temperature (temp), aspartate aminotransferase (AST), Calcium (Ca), Alkaline Phosphatase (ALK), Chloride (Cl), Alanine Aminotransferase (ALT), Creatinine (CRE), Total Carbon Dioxide (tCO2), Albumin (Alb), Blood Urea Nitrogen (BUN), Total Protein (TP), weakness, vomit, edema, confusion, respiratory rate, back pain, dizziness, retrosternal pain, diarrhea, heart rate, diastolic pressure, and abdominal pain. Boxplots and histograms for all factors are depicted in S1 and S2 Figs, which also presents the P-values for the association between Outcome and each factor, for the Fisher exact and T-tests (for nominal and numerical factors, respectively).

**Fig 3:**
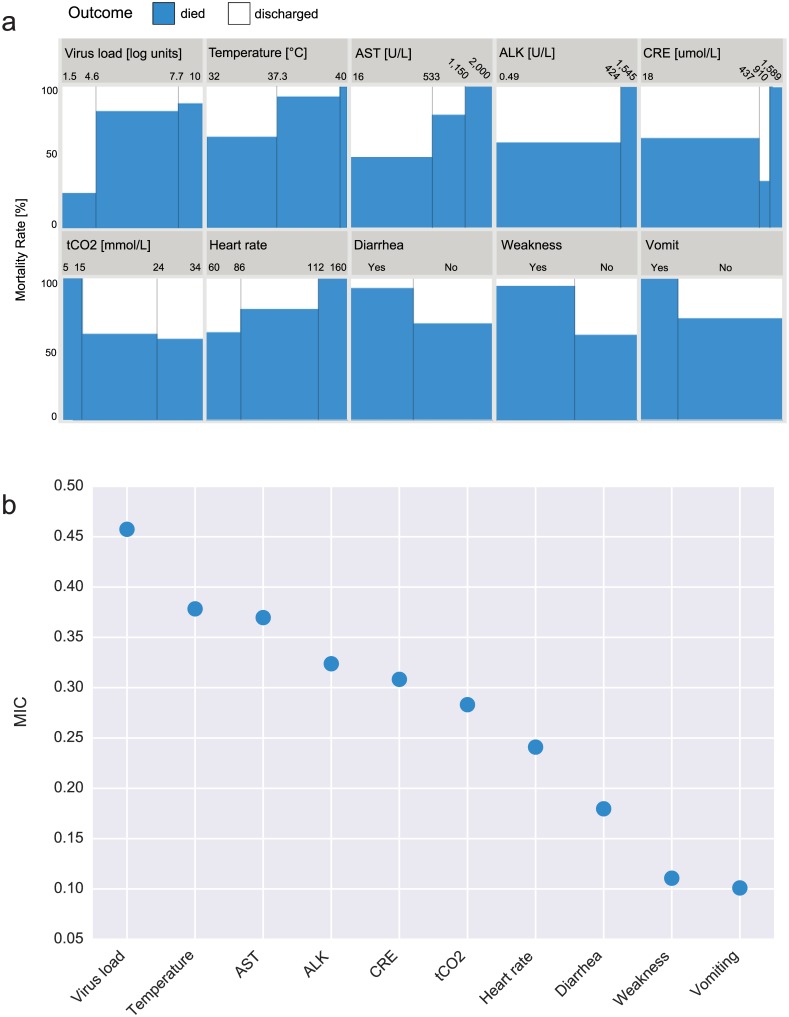
The ten variables that have the highest Mutual Information content with EVD outcome, as ranked with MIC. This set of 10 variables include virus load (PCR), temperature, aspartate aminotransferase (AST), Alkaline Phosphatase (ALK), Alanine Aminotransferase (ALT), Creatinine (CRE), Total Carbon Dioxide (tCO2), heart rate, diarrhea, weakness, and vomit. The plot in panel A represents the eikosograms for all 10 variables, generated from the clinical records of 65 EVD patients between 10 and 50 years of age and known outcome. An eikosogram is a plot that represents the conditional probabilities of one variable (in this case outcome) as a function of the conditioning variable. Staircase shapes in an eikosogram are indicative of association. The plot in panel B shows the ranking of the 10 variables by their MIC score with outcome.

We applied the Maximal Information Coefficient (MIC) statistic developed by Reshef et al. [19], calculated with the MINE program (http://www.exploredata.net/), to rank these 24 factors. We used the ranking to select two informative subsets of 10 variables each (shown in Fig 3), one with PCR and the other without, by picking the top 5 laboratory results and top 5 clinical chart variables. The PCR set comprises PCR, temp, AST, ALK, CRE, tCO2, heart rate, diarrhea, weakness, and vomit, while the non-PCR set includes temp, AST, ALK, CRE, tCO2, BUN, heart rate, diarrhea, weakness, and vomit. None of these variables are capable of predicting outcome accurately in isolation. The performance of the univariate LR classifier is highest with PCR as input, with an F1-score of 0.67, and below 0.5 for all other variables. This result is consistent with the recent report from Crowe et al. [32], which highlights the importance of viral load in the prognosis of EVD.

### Multiple Imputation performance

We evaluated the impact of the MI step on the predictors’ performance, and chose MI parameters accordingly. In all three MI modules, *Amelia II*, MICE and *Hmisc*, we can adjust the fraction of complete records to be included in the data to impute, as well as the number of imputed copies that are aggregated into a single training set. We considered all combinations of these two parameters, when allowing 20%, 35%, and 50% as the percentages of complete records used during imputation, and 1, 5 and 10 for the number of imputed copies. We examined the resulting 9 combinations of parameters across the 4 predictors, LR, ANN, DT, and SVM. Accuracy, as measured by mean F1-score, in the PCR case does not seem to depend on the number of imputed copies, percentage of completed records, and MI algorithm (S3A Fig). In contrast, both higher percentage of completed records and higher number of imputed copies do have a definite enhancing effect in the mean F1-score for the non-PCR case (S3B Fig), while the choice of MI algorithm does not seem to have a significant impact. Counter intuitively, the standard deviation of the F1-score in the PCR case increases with larger percentage of completed records. However, this trend can be explained as follows: the complete records not included in the training set are used to construct the testing set, therefore higher percentages of complete records used during MI result in smaller testing sets. The effect of a single false positive or negative is proportionally larger in smaller testing sets than in larger ones, which results in higher variation of the F1-score in the latter.

We then verified the validity of the MCAR condition in both the PCR and non-PCR sets, crucial to guarantee unbiased imputations, using Little’s chi-square test and Jamshidian and Jalal’s test. Since the all data used in our models was collected at presentation, there is lower risk of non-random missing patterns due to patient death and withdrawal. The tests for MCAR indeed confirm this: Little’s statistic takes a value of 45.28 with a P-value of 0.11 on the PCR set, while the non-PCR set gives a statistic value of 19.06 with a P-value of 0.32, meaning that in both cases there is no evidence in the data against the MCAR hypothesis at the 0.05 significance level. Furthermore, Jamshidian and Jalal’s test for Homoscedasticity, Multivariate Normality, and MCAR does not reject the multivariate normality or MCAR hypothesis at the 0.05 significance level for both the PCR and non-PCR sets, with P-values of 0.79 and 0.06, respectively. This last result in particular validates the use of the *Amelia II* package, which assumes that the data follows a normal distribution.

Based on these findings as well as on a published review from Horton et al. [33], which shows a marginal improvement with *Amelia* over the other MI methods, we chose *Amelia II* as the default MI method. One weakness of the *Amelia II* program is that combinations of variables that are highly collinear might cause the MI computation to fail to converge. We addressed this problem by re-running the MI using either MICE or *Hmisc* when *Amelia* is detected to fail converging more than 5 times. We generated out training sets with 50% of the complete records in the data to impute, and 5 imputed copies for aggregation into a single training set. The performance difference between 5 and 10 imputed copies did not seem large enough to justify the increased computing times.

### Best-performing predictors for EVD prognosis

Having developed and carefully evaluated our models, we demonstrate that we are able to predict EVD prognosis with a mean F1-score of 0.9 or higher, for EVD patients aged 10 to 50. We arrived at this by exhaustively generating two separate ensembles of predictors, one with PCR data and the other without.

**Fig 4:**
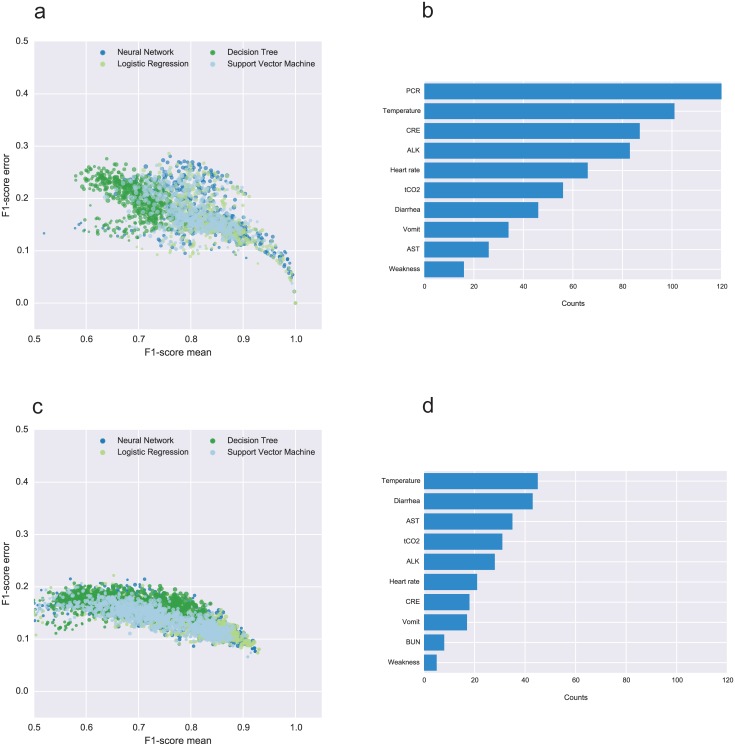
Summary of all models generated with and without PCR data. Each point in scatter plots (a) and (c) represents a predictive model, defined by a particular selection of input variables and a prediction algorithm (LR, ANN, DT, or SVM), trained and tested 100 times. The mean F1-score (weighted average of the precision and sensitivity) calculated over the 100 testing iterations is shown in horizontal axis, while the standard deviation of the F1-score is represented in the vertical axis. The size of the point is proportional to the number of input variables. The bar plots on the right (panels b and d) show the number of times each variable appears in a predictor with mean F1-score above 0.9. Panels A and B represents the models including PCR data, while C and D, represent those without.

The predictors including PCR data are plotted on a scatter plot of the mean F1-score vs standard deviation (Fig 4a) computed over 100 rounds of cross-validation for each predictor. The ensemble consists of 4 × (2^9^–1) = 2044 predictors (LR, ANN, DT, SVM) that were trained on all combinations of the PCR set (9 variables), having PCR as a fixed input variable. The LR and ANN classifiers are the best performers over all the four prediction methods, with 156 models (71 ANN, 64 LR, 21 SVM) yielding an F1-score of 0.9 or higher.

Similarly, we generated 4 × (2^10^–11) = 4052 predictors without PCR data (Fig 4c), which were trained on all combinations of the non-PCR set of variables (10 variables) with at least two elements. We obtained 45 models (18 ANN, 24 LR, 3 SVM) with a mean F1-score of 0.9 or higher. A number of the variables emerged as those most often included in the top-ranked models, both in the PCR and non-PCR cases respectively (Fig 4b and 4d). Notably, in addition to temperature, CRE, ALK, and tCO2 levels are consistently present in the predictors including PCR, while the lack of PCR data makes AST levels and the onset of diarrhea more relevant for accurate prognosis.

**Fig 5:**
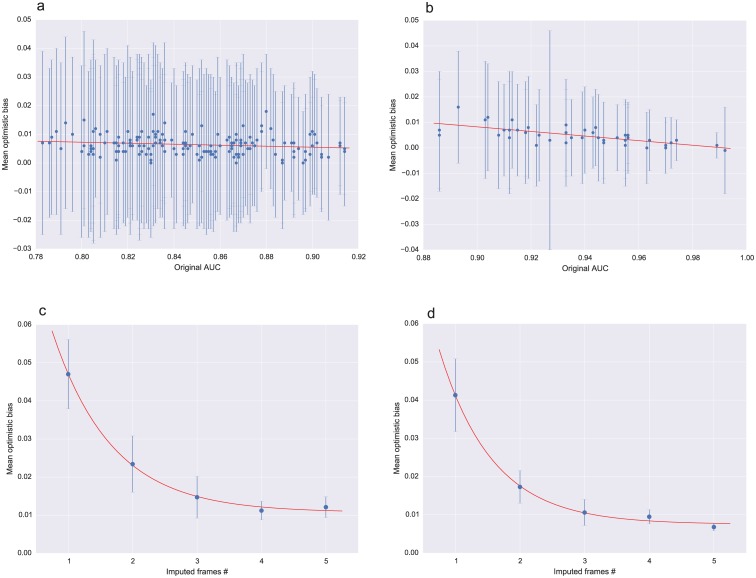
Optimistic-bias estimation. The optimistic bias for the AUC scores of all top PCR (a) and non-PCR (b) predictors were estimated using bootstrap sampling method, averaging the difference between the AUC on the original data and the bootstrap samples over 100 iterations. The scatter plots show the original AUC scores for each model in the horizontal axis, the mean bias on the vertical axis, and the standard deviation of the bias as the error bar. Panels (c) and (d) show the dependency of the optimistic bias as a function of the number of imputed copies, for a logistic regression model that results of applying backward variable selection on the PCR (c) and non-PCR sets of variables (d). The backward selection algorithm was run 10 times for each number of imputed copies, and the mean bias over the 10 iterations is presented, with the standard deviation as the error bars. The bias is quite large when only one imputation is computed, but it decreases exponentially towards 0.01 as the number of multiple imputations increases. The red lines in all plots represent least squares fitted curves, using a linear function in (a, b), and an exponential curve in (c, d), thus highlighting the nature of the dependency of the optimistic bias as a function of the AUC, and the number of imputed copies.

The optimistic bias of the AUC for the top predictors, both in the PCR and non-PCR cases, is below 0.01 for most of them, with a standard deviation of 0.03 (Fig 5a and 5b). This analysis indicates that even though our models are over-fitted for the current data, the magnitude of bias is minor. S1 and S2 Tables detail all the top-performing predictors and their optimism-corrected AUC scores in the PCR and non-PCR cases, respectively. S5 Fig shows aggregated ROC curves over all the models for each predictor, for the PCR and non-PCR cases. The aggregated AUCs are 0.96 (LR), 0.95 (ANN), 0.94 (SVM), and 0.84 (DT) in the PCR models, and 0.88 (LR, ANN), 0.86 (SVM), and 0.77 (LR) in the non-PCR models. The similar performance of our simple ANN predictor and scikit-learn’s LR classifier suggests that the dependency between the covariates and outcome can be modeled linearly, however larger datasets would enable us to train more complex ANNs with potentially better performance across different groups of patients.

The comparison with variable selection shows an effect of the MI protocol similar to that observed in the top-ranked models. The optimistic bias of the AUC for the selected PCR and non-PCR models consistently decreases to less than 0.01 as the number of imputed copies increases from 1 to 5 (Fig 5c and 5d). On the other hand, these models assign very small coefficients and odd ratios very close to 1 to the laboratory covariates (Tables 1 and 2). This suggests that most of the information in these models is captured by the clinical symptoms (temperature, diarrhea, vomit), although weakness consistently presents an odd ratio less than 1, contradicting the expected dependency with outcome. In general, the laboratory variables are the highest ranked according to MIC, and are also included in most of the top-ranked models, using either the LR or ANN classifiers. These results lead us think that the variable selection approach is discarding relevant information for outcome prediction, which we are able to capture in our ensemble of ML predictors.

**Table 1:** Logistic Regression coefficients and odd ratios for the PCR set. This table shows the coefficients of the logistic regression model that results from backward variable selection with AIC, applied on the PCR and non-PCR sets. The coefficients of each model are together with the P-value of the Wald test with a null hypothesis consisting of the maximum likelihood estimate of the coefficient being equal to zero, the odd-ratios for a unit change in each variable, and their 95% confidence interval.

Variable	Coefficient	P-value	Odds ratio	95% CI
**PCR**	4.815×10^−2^	2.36×10^−7^	1.049	[1.030, 1.068]
**Temperature**	1.365×10^−1^	1.49×10^−13^	1.146	[1.106, 1.187]
**CRE**	-2.155×10^−4^	5.58×10^−5^	0.999	[0.9997, 0.9999]
**tCO2**	-2.943×10^−2^	< 2×10^−16^	0.971	[0.965, 0.976]
**Heart rate**	-1.641×10^−3^	0.0165	0.998	[0.997, 0.999]
**Diarrhea**	4.577×10^−1^	< 2×10^−16^	1.580	[1.474, 1.694]
**Weakness**	-2.588×10^−1^	1.58×10^−13^	0.772	[0.722, 0.825]
**Vomit**	2.884×10^−1^	< 2×10^−16^	1.334	[1.248, 1.426]

**Table 2:** Logistic Regression coefficients and odd ratios for the non-PCR set. This table shows the coefficients of the logistic regression model that results from backward variable selection with AIC, applied on the non-PCR sets. The interpretation of coefficients, P-values, odd-ratios and confidence intervals is the same as in Table 1.

Variable	Coefficient	P-value	Odds ratio	95% CI
**Temperature**	2.420×10^−1^	< 2×10^−16^	1.274	[1.237, 1.311]
**CRE**	2.324×10^−4^	0.038449	1.0002	[1.00001, 1.0004]
**tCO2**	-2.392×10^−2^	< 2×10^−16^	0.976	[0.971, 0.981]
**BUN**	-8.988×10^−3^	0.000879	0.991	[0.986, 0.996]
**Diarrhea**	4.628×10^−1^	< 2×10^−16^	1.588	[1.497, 1.686]
**Weakness**	-2.080×10^−1^	3.76×10^−11^	0.812	[0.767, 0.863]
**Vomit**	3.063×10^−1^	< 2×10^−16^	1.358	[1.279, 1.443]

### Performance of prognosis app

The *Ebola CARE* app packages a total of 82 ANN models, selected from those with a mean F1-score above 0.9, but discarding the models with a standard deviation of 0, in order to avoid potentially overfitted models. This set incorporates 64 PCR and 18 non-PCR models, so the app can still be used when viral load information is not available. We entered into *Ebola CARE* all the patients who had complete data for at least one model in the app, and recorded the risk prediction as presented after inputting the symptoms. Predictions for a total of 34 patients were obtained in this way. For this subgroup of patients, the mortality rate was 79% (7 survived, 27 died), and the app only misclassified two, one in each outcome group. In other words, the precision and sensitivity were both 0.96. However, this number is likely overestimating the performance of the app, since some of these patients used in this test were also included in model training.

### Data and source code availability

The data used in this study is hosted at a Dataverse in the Harvard Dataverse Network (http://dx.doi.org/10.7910/DVN/29296), the source code of *Mirador* and the ML pipeline is available on Github (https://github.com/mirador/mirador, https://github.com/broadinstitute/ebola-predictor), and the model files (all training and testing sets) are deposited on Zenodo (http://dx.doi.org/10.5281/zenodo.19831).

## Discussion

This work represents the first known application of ML techniques to EVD prognosis prediction. The results suggest that a small set of clinical symptoms and laboratory tests could be sufficient to accurately prognosticate EVD outcome, and that these symptoms and tests should be given particular attention by health care professionals. By aggregating all the high-performing models obtained in our exhaustive analysis, we can construct a composite algorithm that runs the best predictor depending on the available data. We have developed a simple app, *Ebola CARE*, which can be installed on mobile tablet or phone devices, and would complement rapid EVD diagnostic kits and data-entry devices.

Our *Ebola CARE* app is a proof-of-concept, only applicable to Ebola Zaire patients treated in similar conditions as those in KGH. New clinical data will enable us and other groups to independently validate the app, and to generate more generalizable models with higher statistical significance. Within the current constrains, the results also shed light on the most informative clinical predictors for adult patients -temperature, diarrhea, creatinine, alkaline phosphatase, aspartate aminotransferase, total carbon dioxide- and demonstrate that PCR provides critical additional information to quantify the seriousness of the Ebola virus infection and better estimate the risk of the patients. In general, these results are consistent with the findings from Schieffelin, Levine, Yan, and Zhang. Current discrepancies–for instance Zhang reports chest pain, coma, and confusion as significantly associated with EVD prognosis whereas we do not–could be attributed to the small sample sizes, missing data, and different clinical protocols at the various treatment sites.

The prevalence of missing data in the dataset used in this study, and the lack of other publicly available datasets, are fundamental challenges in predictive modeling. By combining MI with four distinct ML predictors, we offer a direct approach for dealing with the first challenge. The use of ANN and LR classifiers in combination with a MI enrichment methodology shows promise as a way to accurately predict outcome of EVD patients given their initial clinical symptoms and laboratory results.

New patient data is critical to validate and extend these results and protocols. Richer datasets incorporating more diverse samples from different locations will allow us and other researchers to train better ML classifiers and to incorporate population variability. The development of survival models could be another very important application of these techniques to assist not only in prognosis upon patient intake but also during treatment, as shown by Zhang. Our current data includes time courses that would be useful in this kind of models, but unfortunately only for a handful of patients. All these facts highlight the importance of immediate availability of clinical data in the context of epidemic outbreaks, so that accurate predictive tools can be quickly adopted in the field.

In summary, we have made our protocol and mobile app publicly available, fully documented (https://github.com/broadinstitute/ebola-predictor/wiki), and readily adaptable to facilitate and encourage open data sharing and further development. Our integration of *Mirador*, a tool for visual exploratory analysis of complex datasets, and an ML pipeline defines a complete framework for data-driven analysis of clinical records, which could enable researchers to quickly identify associations and build predictive models. Our app is similarly designed to be easily updated as new predictive models are developed with our pipeline, validated with better data, and packaged, to generate actionable diagnosis and help inform urgent clinical care in outbreak response.

## Supporting Information

S1 Fig Boxplots of numerical clinical factors identified with Mirador. Boxplots of all numerical variables (PCR, temperature, AST, CRE, ALT, ALK, BUN, tCO2, heart rate, Alb, Ca, TP, Cl, respiratory rate, and diastolic pressure). Separate boxplots are generated for the discharged and deceased patients. The P-value on top of each plot corresponds to the T-test for different means of two independent samples, as evaluated with the ttest_ind() function in SciPy (http://docs.scipy.org/doc/scipy/reference/generated/scipy.stats.ttest_ind.html).
(EPS)

S2 Fig Histograms of nominal clinical factors identified with Mirador. Histograms for all the categorical variables (weakness, diarrhea, dizziness, vomit, confusion, edema, abdominal pain, back pain, and retrosternal pain). These histograms illustrate the fraction of the patients affected by the condition both for those who were discharged and deceased. The P-value on top of each plot corresponds to the Fisher exact test on the 2x2 contingency table for the symptom variable vs outcome, as evaluated with the fisher_exact() function in SciPy (http://docs.scipy.org/doc/scipy/reference/generated/scipy.stats.fisher_exact.html).
(EPS)

S3 Fig Effect of number of imputed copies, percentage of complete records, and imputation algorithm. Each panel shows the scatter plot matrix for all combinations of number of imputed copies (1, 5, 10) and percentage of completed records used in imputation (20%, 35%, 50%). Panel A was generated from predictions on three PCR models -(i) PCR, temp, heart rate, (ii) PCR,temp, AST, vomit, (iii) PCR, temp, ALK, tCO2, weakness- and panel B from three non-PCR models -(i) temp, BUN, vomit, (ii) temp, AST, tCO2, vomit, (iii) temp, CRE, tCO2, diarrhea, weakness.
(EPS)

S4 Fig Scatter plot matrix showing training set before and after imputation. Scatter plot matrices where the test data for the model defined by the variables PCR temperature, and AST. The matrix on the top includes only the complete records present in the training set (instances with at least one missing value are not drawn). Plots off the diagonal represent the scatter plot between each combination of variables, while the plots along the diagonal contain the histograms for each variable computed from the available data. The *Amelia* procedure described in the text generates 5 imputed copies of the incomplete training set, where each missing entry is replaced by a value sampled from the distribution inferred from the observed data. Since the entire training set had 60 records (although only 8 were complete), each imputed frame contains 60 complete records, which were aggregated to result in a training set with 300 elements.
(EPS)

S5 Fig ROC curves for the PCR and non-PCR models. The ROC curves were obtained by aggregating all models, grouped by ML algorithm, and offer a picture of the overall performance of the LR, ANN, DT, and SVM algorithm on all possible combination of input variables. Panel A corresponds to the models including PCR data, and panel C to those without. The AUCs of these curves are 0.96 (LR), 0.95 (ANN), 0.94 (SVM), and 0.84 (DT) in the PCR models, and 0.88 (LR, ANN), 0.86 (SVM), and 0.77 (LR) in the non-PCR models.
(EPS)

S1 Table AUC for top-ranked models including PCR. This table shows all models including the PCR variable as input and having a mean F1-score above 0.9 (total of 156). LR, ANN, and SVM models were included, but no DT as all of them have a mean F1-score below 0.9). The original AUC computed over the entire sample, the mean and standard deviation optimistic bias (calculated using Harrell’s bootstrap method), and the corrected AUC (defined as the original AUC minus the mean bias) are shown for each model.
(DOCX)

S2 Table AUC for top-ranked models not including PCR. This table shows all models without the PCR variable as input and having a mean F1-score above 0.9 (total of 45). As in S1 Table, LR, ANN, and SVM models were included, but no DT models reached the 0.9 threshold. The original AUC computed over the entire sample, the mean and standard deviation optimistic bias (calculated using Harrell’s bootstrap method), and the corrected AUC (defined as the original AUC minus the mean bias) are shown for each model.
(DOCX)
