# Supplementary material for: Transforming Clinical Data into Actionable Prognosis Models: Machine-Learning Framework and Field-Deployable App to Predict Outcome of Ebola Patients
Source: PLoS Negl Trop Dis. 2016 Mar 18;10(3):e0004549. doi: 10.1371/journal.pntd.0004549 (PMC4798608; doi:10.1371/journal.pntd.0004549)
Supplement: S2 Table — This table shows all models without the PCR variable as input and having a mean F1-score above 0.9 (total of 45). As in S1 Table, LR, ANN, and SVM models were included, but no DT models reached the 0.9 threshold. The original AUC computed over the entire sample, the mean and standard deviation optimistic bias (calculated using Harrell’s bootstrap method), and the corrected AUC (defined as the original AUC minus the mean bias) are shown for each model. (DOCX) [file pntd.0004549.s007.docx]

**Table S2**

| Predictor | Variables | Original AUC | Mean bias | STD bias | Corrected AUC |
| --- | --- | --- | --- | --- | --- |
| LR | Temperature, AST, ALK, Heart rate, Diarrhea | 0.93 | 0.01 | 0.02 | 0.93 |
| LR | Temperature, AST, Heart rate, Diarrhea | 0.95 | 0.00 | 0.01 | 0.94 |
| ANN | Temperature, AST, Heart rate, Diarrhea | 0.95 | 0.00 | 0.02 | 0.94 |
| ANN | Temperature, AST, ALK, Heart rate, Diarrhea | 0.94 | 0.00 | 0.01 | 0.93 |
| LR | Temperature, AST, ALK, CRE, tCO2, Heart rate, Diarrhea, Weakness, Vomit | 0.90 | 0.01 | 0.02 | 0.89 |
| ANN | Temperature, AST, ALK, CRE, tCO2, Heart rate, Diarrhea, Weakness, Vomit | 0.90 | 0.01 | 0.02 | 0.89 |
| LR | Temperature, AST, tCO2, Heart rate, Diarrhea | 0.96 | 0.00 | 0.01 | 0.95 |
| ANN | Temperature, tCO2, Diarrhea | 0.97 | 0.00 | 0.01 | 0.97 |
| SVM | Temperature, Diarrhea | 0.93 | 0.00 | 0.04 | 0.92 |
| LR | Temperature, tCO2, Diarrhea | 0.97 | 0.00 | 0.01 | 0.97 |
| ANN | Temperature, AST, tCO2, Heart rate, Diarrhea | 0.96 | 0.01 | 0.01 | 0.95 |
| LR | Temperature, AST, ALK, tCO2, Heart rate, Diarrhea | 0.99 | 0.00 | 0.01 | 0.99 |
| LR | Temperature, AST, ALK, tCO2, Diarrhea, Vomit | 0.92 | 0.01 | 0.02 | 0.91 |
| LR | Temperature, AST, tCO2, Diarrhea | 0.89 | 0.01 | 0.02 | 0.88 |
| ANN | Temperature, AST, ALK, tCO2, Heart rate, Diarrhea | 0.99 | 0.00 | 0.01 | 0.99 |
| LR | Temperature, AST, ALK, CRE, tCO2, BUN, Heart rate, Diarrhea, Vomit | 0.92 | 0.01 | 0.02 | 0.91 |
| LR | Temperature, AST, ALK, CRE, tCO2, Heart rate, Diarrhea | 0.97 | 0.00 | 0.01 | 0.97 |
| LR | Temperature, AST, CRE, tCO2, Heart rate, Diarrhea | 0.94 | 0.00 | 0.02 | 0.94 |
| ANN | Temperature, AST, ALK, CRE, tCO2, Diarrhea | 0.94 | 0.01 | 0.02 | 0.93 |
| SVM | Temperature, Diarrhea, Vomit | 0.99 | -0.00 | 0.02 | 0.99 |
| ANN | Temperature, AST, ALK, tCO2, Diarrhea, Vomit | 0.92 | 0.01 | 0.02 | 0.91 |
| ANN | Temperature, AST, tCO2, Diarrhea | 0.89 | 0.01 | 0.02 | 0.88 |
| LR | Temperature, AST, ALK, CRE, tCO2, Diarrhea | 0.94 | 0.00 | 0.02 | 0.94 |
| LR | Temperature, ALK, tCO2, Diarrhea | 0.95 | 0.00 | 0.02 | 0.95 |
| LR | Temperature, AST, ALK, CRE, tCO2, BUN, Diarrhea, Vomit | 0.94 | 0.01 | 0.01 | 0.94 |
| ANN | Temperature, AST, ALK, CRE, tCO2, Heart rate, Diarrhea | 0.97 | 0.00 | 0.01 | 0.97 |
| ANN | Temperature, ALK, tCO2, Diarrhea | 0.96 | 0.00 | 0.01 | 0.95 |
| LR | Temperature, AST, ALK, Heart rate, Diarrhea, Vomit | 0.95 | 0.01 | 0.01 | 0.95 |
| ANN | Temperature, AST, Diarrhea | 0.91 | 0.01 | 0.02 | 0.91 |
| LR | Temperature, AST, CRE, Heart rate, Diarrhea | 0.91 | 0.01 | 0.02 | 0.90 |
| LR | Temperature, AST, ALK, CRE, Heart rate, Diarrhea | 0.92 | 0.01 | 0.02 | 0.91 |
| LR | Temperature, AST, Diarrhea | 0.91 | 0.00 | 0.02 | 0.91 |
| ANN | Temperature, AST, ALK, CRE, tCO2, BUN, Heart rate, Diarrhea, Vomit | 0.91 | 0.01 | 0.02 | 0.90 |
| LR | Temperature, AST, Heart rate, Diarrhea, Vomit | 0.93 | 0.01 | 0.02 | 0.93 |
| ANN | Temperature, AST, ALK, CRE, tCO2, BUN, Diarrhea, Vomit | 0.94 | 0.01 | 0.01 | 0.94 |
| LR | Temperature, ALK, CRE, tCO2, Diarrhea | 0.96 | 0.00 | 0.01 | 0.96 |
| SVM | Temperature, AST, ALK, CRE, tCO2, Heart rate, Diarrhea, Weakness, Vomit | 0.89 | 0.02 | 0.02 | 0.88 |
| LR | Temperature, AST, ALK, CRE, tCO2, BUN, Diarrhea | 0.92 | 0.00 | 0.02 | 0.92 |
| LR | Temperature, tCO2, Vomit | 0.93 | 0.00 | 0.02 | 0.93 |
| ANN | Temperature, AST, Heart rate, Diarrhea, Vomit | 0.93 | 0.01 | 0.02 | 0.92 |
| LR | Temperature, ALK, Vomit | 0.91 | 0.01 | 0.02 | 0.90 |
| ANN | Temperature, ALK, CRE, tCO2, Diarrhea | 0.96 | 0.00 | 0.01 | 0.96 |
| LR | Temperature, AST, ALK, tCO2, BUN, Diarrhea, Weakness, Vomit | 0.95 | 0.00 | 0.01 | 0.95 |
| ANN | Temperature, AST, ALK, CRE, tCO2, BUN, Diarrhea | 0.92 | 0.01 | 0.02 | 0.92 |
| ANN | Temperature, AST, ALK, tCO2, BUN, Diarrhea, Weakness, Vomit | 0.95 | 0.00 | 0.01 | 0.95 |
